# The Chemical Space of Terpenes: Insights from Data Science and AI

**DOI:** 10.3390/ph16020202

**Published:** 2023-01-29

**Authors:** Morteza Hosseini, David M. Pereira

**Affiliations:** REQUIMTE/LAQV, Laboratório de Farmacognosia, Departamento de Química, Faculdade de Farmácia, Universidade do Porto, R. Jorge Viterbo Ferreira, 4050-313 Porto, Portugal

**Keywords:** terpenes, natural products, data science, big data, chemometrics, classification

## Abstract

Terpenes are a widespread class of natural products with significant chemical and biological diversity, and many of these molecules have already made their way into medicines. In this work, we employ a data science-based approach to identify, compile, and characterize the diversity of terpenes currently known in a systematic way, in a total of 59,833 molecules. We also employed several methods for the purpose of classifying terpene subclasses using their physicochemical descriptors. Light gradient boosting machine, k-nearest neighbours, random forests, Gaussian naïve Bayes and Multilayer perceptron were tested, with the best-performing algorithms yielding accuracy, F1 score, precision and other metrics all over 0.9, thus showing the capabilities of these approaches for the classification of terpene subclasses. These results can be important for the field of phytochemistry and pharmacognosy, as they allow the prediction of the subclass of novel terpene molecules, even when biosynthetic studies are not available.

## 1. Introduction

The natural products chemical space is a fascinating set of hundreds of thousands of molecules that are remarkably interesting not only from a strictly chemical point of view, but also owing to the diverse and impressive set of biological properties that many of these molecules possess. The importance of this chemical space is further evidenced by the significant number of such molecules that are currently used as medicines in human and veterinary medicine [1].

From a phytochemical and pharmacognostic point of view, natural molecules are grouped into different families on the grounds of their biosynthetic origin, or sometimes due to their shared chemical traits [2].

Many families of natural molecules are of interest for human health, not only given their role in medicine, but also given their application in nutrition or cosmetics. Among such families, terpenes are a diverse set of compounds that have paved their way as medicines (artemisinin, taxol, among others), but also in industries as diversified as foodstuffs (carotenoids as colouring agents), flavours (menthol, limonene, and pinene) and preservatives (eugenol).

Given the tens of thousands of terpenes known, they are frequently grouped on the grounds of their number of carbons, which in turn reflects their biosynthetic approach [2]. Briefly, depending on the number of C_5_ isoprene units, terpenes can be monoterpenes (C_10_), sesquiterpenes (C_15_), diterpenes (C_20_), triterpenes (C_30_) and so on. Some of these molecules can also be lactones or can bear sugars, thus being routinely classified as terpene lactones or terpene glycosides, respectively.

Like other classes of natural products, the fast pace at which new molecules are described makes it increasingly difficult to continue to manually curate and study the chemical descriptors of each molecule individually. To this end, data science-based approaches are needed, as they allow organizing, interpreting, and filtering huge amounts of data and, sometimes, highlight relationships that would otherwise eclipse human attention.

As it is frequently postulated, the quality of the data in data science-centred analysis is of paramount importance. Fortunately, the digitalization of information has enabled data science-based frameworks to study the natural products chemical space.

In this work, the authors aimed to employ a data science and artificial intelligence approach to the largest and most complete database of natural products to date, the COCONUT database [3]. The latest reference we could find stated that over 55,000 terpenes were known [4], and now the authors included 59,833 terpenes from this resource and employed a data science approach to extract, describe and interpolate data from these data sources. The result is a compendium of information on terpenes, on a subclass-by-subclass basis, that can be a valuable resource for researchers, teachers and students of this exciting branch of natural products chemistry and pharmacognosy. As a proof of concept, the authors employed several algorithms to cluster and classify these molecules solely on the grounds of their physio-chemical properties.

## 2. Results

### 2.1. Defining the Chemical Space Occupied by Terpenes

The authors’ primary source of data was the COCONUT database, created and curated by the Institute for Inorganic and Analytical Chemistry, Friedrich-Schiller University, Germany [3]. This resource, first made available in 2021, is an online repository of nearly half a million natural products, annotated with several types of physicochemical and topographical information, which compiles the information of over 50 databases of natural molecules. Previous works on terpenes have used commercial databases [5]; however, the authors believe that a public resource such as the one we describe herein is a positive trend towards the democratization of data and science as a whole. Furthermore, previous works have provided 11 physicochemical properties for their dataset [5], while we provide over 40 here.

Starting from the COCONUT database (400k+ molecules), the authors initially created a subset comprising solely the chemical SuperClass “Lipids and lipid-like molecules”, which comprises all molecules of lipidic nature, in which terpenes are included. Among this subset (99,696 molecules, distributed according to Supplementary Table S1), the authors further selected the classes “Monoterpenes, “Sesquiterpenes”, “Diterpenes”, “Sesterterpenes”, “Triterpenes”, “Polyterpenes”, “Sesquaterpenes”, “Terpene glycosides” and “Terpene lactones” in order to include all terpene classes. This yielded the final dataset, in which are included 59,833 molecules. The authors acknowledge that many molecules in the “Terpene glycosides” and “Terpene lactones” subclasses can be, from a phytochemical point of view, part of any of the remaining subclasses. However, for the sake of clarity and access to as many molecules as possible, the authors followed the hierarchical criteria enforced by COCONUT.

**Figure 1:**
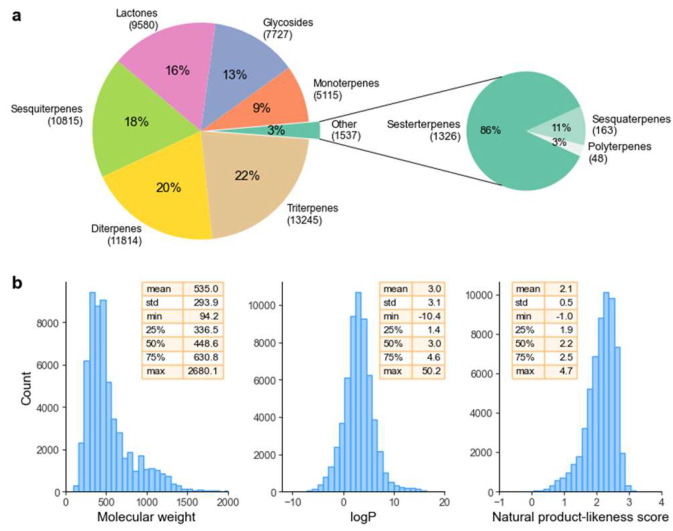
(**a**) Overview of absolute and relative counts of terpene subclasses. (**b**) Distribution of molecular weight, natural product-likeness score and logP for all terpenes in the dataset. For the sake of clarity, we have chosen only molecular weights up to 2000 and −12 < logP < 20, with only 0.2% and 0.1% of the molecules being suppressed, respectively.

Figure 1 depicts the distribution of the molecules in the dataset across different terpene classes. As shown, the major classes were triterpenes (22.1%, 13,245 molecules), followed by diterpenes (19.7%, 11,814 molecules), and the least expressive class was polyterpenes (0.1%, 48 molecules). Before advancing, it is important to briefly review the organization of COCONUT in terms of chemical ontology. 

**Figure 2:**
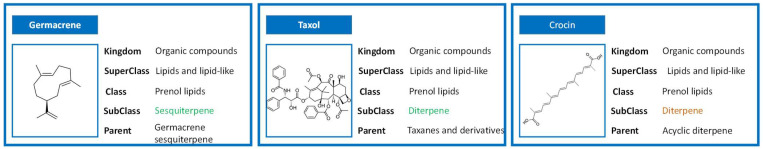
Chemical classification of germacrene, taxol and crocin according to ClassyFire.

After a registry is created, several parameters are automatically calculated based on well-established algorithms. For example, in the case of the chemical classification, Classyfire is used [6]. This is a remarkable resource, as this is a purely structure-based chemical taxonomy software that uses chemical structures and structural features as inputs to assign a given molecule to a hierarchical-based taxonomy. This new chemical taxonomy consists of up to 11 different levels (including Kingdom, SuperClass, Class, SubClass, etc.), with each of the categories defined by unambiguous and computable structural rules. This approach is both powerful and dangerous in the case of the natural products chemical space. On the one hand, it is possible to easily classify hundreds of thousands of molecules based on clearly established rules, instead of having to rely in biosynthetic studies. As an example, when we run taxol into Classifyre, we get the classification: Kingdom: Organic compounds > Superclass: Lipids and lipid-like > Class: Prenol Lipids > Subclass: Diterpene > Classification: Taxanes and derivatives (Figure 2). From a phytochemical point of view, one would not usually include terpenes in the class of lipids (older textbooks would, however), but it is true they are of lipidic nature, so there is no surprise here. The most important levels of taxonomical classification for a natural products chemist are Subclass: Diterpenes and Parent: Taxanes. This works fine, as they can get both the natural product family and its nucleus/immediate family. To be able to have this degree of information, even in cases where biosynthetic studies are not yet available, is remarkable.

On the other hand, the disadvantage of this rule-based classification is that natural products are a wonderful source of rule breakers and deviants. Take, for instance, crocin, from *Crocus sativus*. From a phytochemical point of view, crocin is a (di)apocarotenoid, as its loss of isoprene units from both ends renders it a chemical entity with C_20_. Naturally, a rule-based algorithm has no way to know that from a biosynthetically point of view, this molecule is a carotenoid (typically C_40_) that has lost carbons. As so, it is classified as “acyclic diterpenoid” (compounds comprising four consecutive isoprene units that do not contain a cycle, Figure 2).

**Figure 3:**
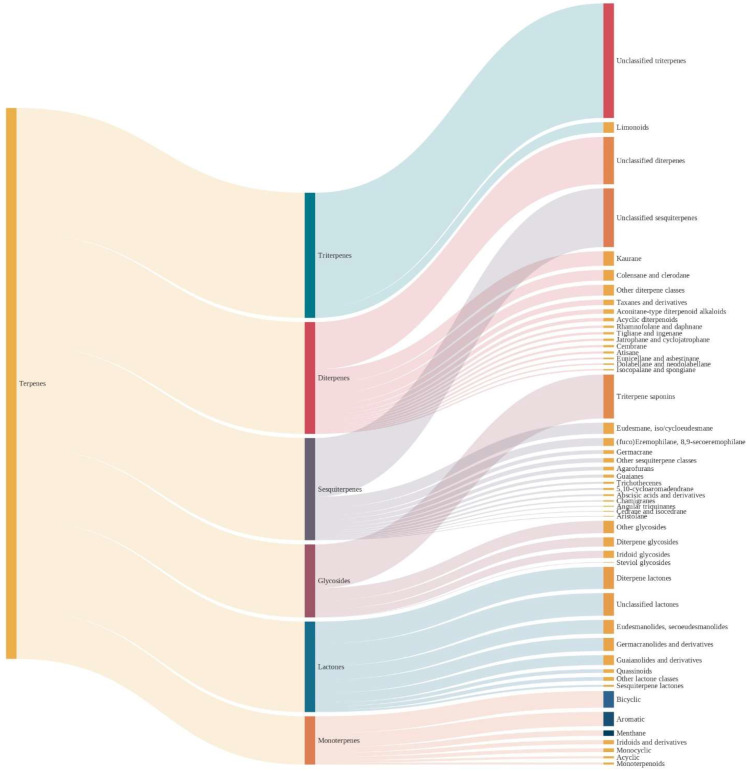
Classification of terpenes in the COCONUT database. The second level of classification corresponds to classical phytochemical subclasses (monoterpenes, diterpenes, sesquiterpenes, among others), while the last level corresponds to characteristic nuclei or subsets.

Nevertheless, despite these limitations, it is helpful to have a tool to quickly classify millions of molecules, with the advances in technology allowing to fine-tune the rules towards increasingly accurate performances. However, some caution must be taken for structures that may be incorrectly classified. In Figure 3, the authors present the different levels of chemical taxonomy for the dataset used in this work.

**Figure 4:**
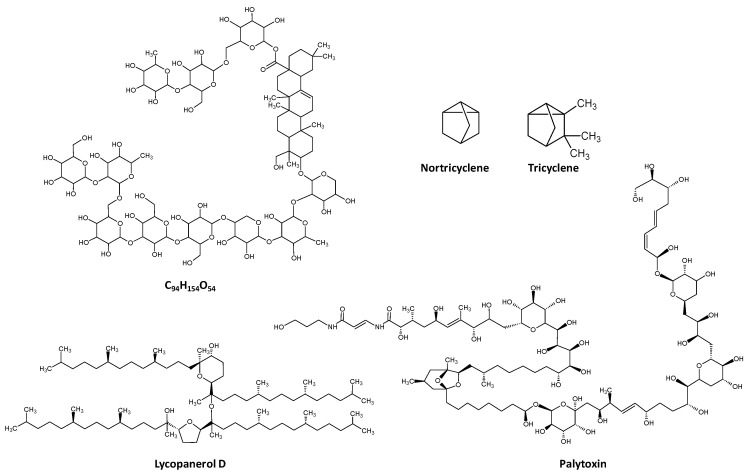
Structure of the terpenes that occupy the lower and upper range of the MW and logP in the terpene family. Nortricyclene is the smallest molecule in the database (94.15 Da), while palytoxin is the one with higher MW (2680.14 Da). The unnamed molecule (no common name) is the entry with the lowest logP (−10.4), while lycopanerol D presents the highest calculated logP (50.2).

Considering all terpenes studied here, the average molecular weight was 534.9 Da (min: 94.2, nortricyclene and max 2680.1, palytoxin, Figure 4). 

Nortricyclene does not comply with the standard monoterpene definition given its odd number of carbons, 7; however, this classification arises from its structural relation to tricyclene (C_10_H_16_, Figure 4). Palytoxin, from Palythoa corals, is one of the largest non-polymeric natural products described and one of the most poisonous non-protein molecules known, second only to maitotoxin in terms of mice toxicity [7]. It is a terpene glycoside and displays 8 double bonds, 40 hydroxyl groups and 64 chiral centres, which gives rise to over 1021 possible stereoisomers.

**Figure 5:**
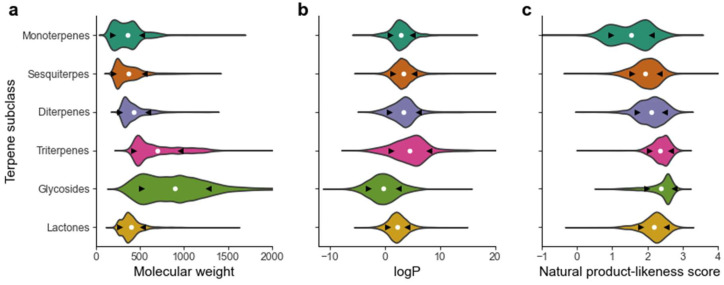
Distribution of (**a**) molecular weight, (**b**) logP and (**c**) natural product-likeness score across different terpenes subclasses. White mark (•) represents the mean. Left black arrow represents the mean—std and the right arrow the mean + std.

The distribution of MW across all terpenes is presented in Figure 1. From a phytochemical point of view, the different terpene subclasses are grouped based on their biosynthesis and, consequently, the number of isoprene units in their structure. For this reason, it is obvious that marked differences in molecular weight can be found across distinct subclasses. For the sake of compiling the molecular weight intervals in each terpene class, Figure 5a shows the molecular weight distribution as a function of the subclass. 

The average molecular weight increases with the increasing number of carbons in the monoterpene < sesquiterpene < diterpene < triterpene order. Terpene glycosides present the widest range of molecular weight, an expected consequence of the occurrence of a different number of sugars and their diverse identity.

The partition coefficient (P), which describes the suitability of a neutral molecule to dissolve in immiscible biphasic systems comprised of lipids and water, is a pivotal characteristic that has a marked impact on the “druggability” of a compound, as extreme values, either too negative or too positive, can hinder its adequate pharmacokinetics in the human body. For example, a study comprising a dataset of 4 leading pharmaceutical companies showed that among 812 molecules studied as drug candidates, the average logP was 3.2. As is shown in Figure 1, the average logP of terpenes is approximately 3, with a molecule with formula C_94_H_154_O_54_ (no common name) and the tetraterpene ether lycopanerol H being the molecules in the lower and upper limit (−10.3 and 50.2, respectively, Figure 4). 

As shown in the class-by-class analysis (Figure 5b), the average value increased in the monoterpene < sesquiterpene < diterpene < triterpene order, the latter being the subclass of terpenes with the highest average logP, 4.6. Terpene glycosides was the only class presenting a negative average logP, as expected given their heterosidic nature that greatly increases their polarity.

The natural product-likeness score is a descriptor that takes into account several structural and topographical features and tries to quantify the “degree of naturalness” of a given molecule [8]. Naturally occurring molecules, owing to their stereochemical complexity and diversified ring systems, usually afford higher values, whereas compounds arising from synthetic chemistry display lower values. As it is shown in Figure 5c, the average value for terpenes was 2.1. Monoterpenes, the simplest class from a structural point of view, had the lowest average natural product-likeness score, 1.5, owing to their simpler chemical structures. This value increases with the chemical complexity of the subclasses in the monoterpene > sesquiterpene > diterpene > triterpene order.

Several terpenes are currently being used in the drugs’ arsenal against a number of diseases. This includes, for example, paclitaxel for cancer, artemisinin for malaria and manoalide for inflammatory processes.

In the process of pharmaceutical development, it is important to take into account the “drug-likeness” of a given molecule in order to identify unlikely candidates as upstream in the development process as possible. One of the most widely spread set of “rules of thumb” are the Lipinski’s rule of five [9], which postulate that ideal drugs would have a number of hydrogen bond donors ≤ 5, number of hydrogen bond acceptors ≤ 10, molecular mass ≤ 500 Da and logP ≤ 5. Although this set of anecdotal rules was postulated a long time ago, most of its basis is still widely used today: in a study with over 800 drug candidates, only 24% had a molecular weight > 500 Da and only 15% of the candidates had a logP > 5. Notably, only 7.6% of all the molecules violated both rules simultaneously [10].

**Figure 6:**
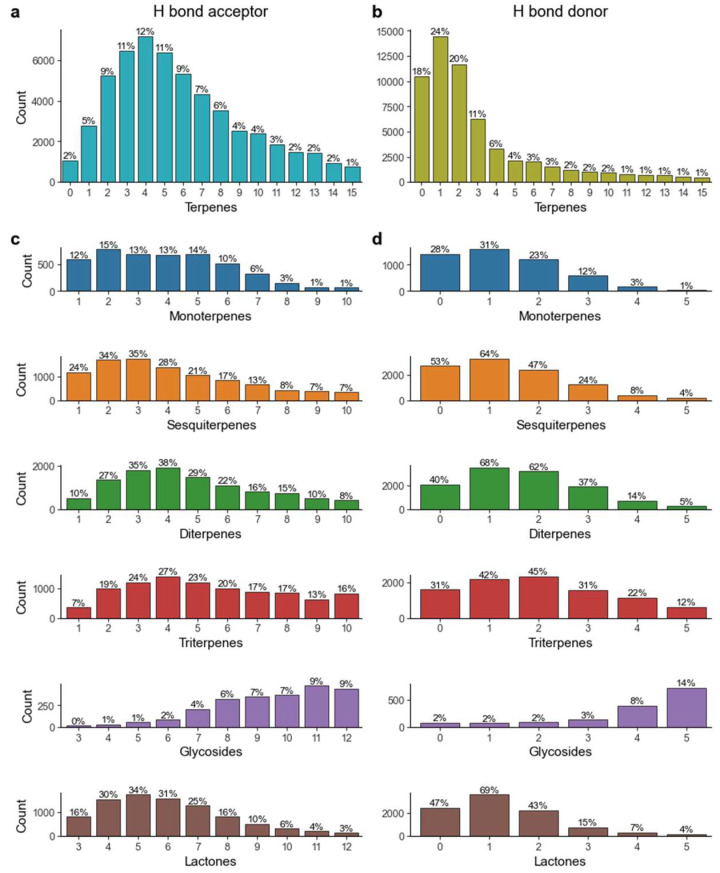
Number of hydrogen bond (**a**) acceptors and (**b**) donors in the terpene chemical space and on a subclass basis for (**c**) acceptors and (**d**) donors.

We also investigated the rate of violation of the terpenes chemical space in terms of the number of hydrogen bond donors and acceptors. As is shown in Figure 6, 78.7% of all terpenes had 10 hydrogen bond acceptors or less, while 81% had 5 hydrogen bond donors or less, thus complying with Lipinski’s rule of five. When studying these parameters on a subclass level, we see that in a general way, monoterpenes, sesquiterpenes, diterpenes and terpene lactones all present over 85% of molecules with 10 or less hydrogen bond acceptors and 70% of molecules with 5 or less hydrogen bond donors. Triterpenes are slightly different, as they exhibit 70% and 59% for the same parameters, respectively. The most distinct set of molecules are terpene glycosides, in which only 18.7% of molecules observe these rules.

**Figure 7:**
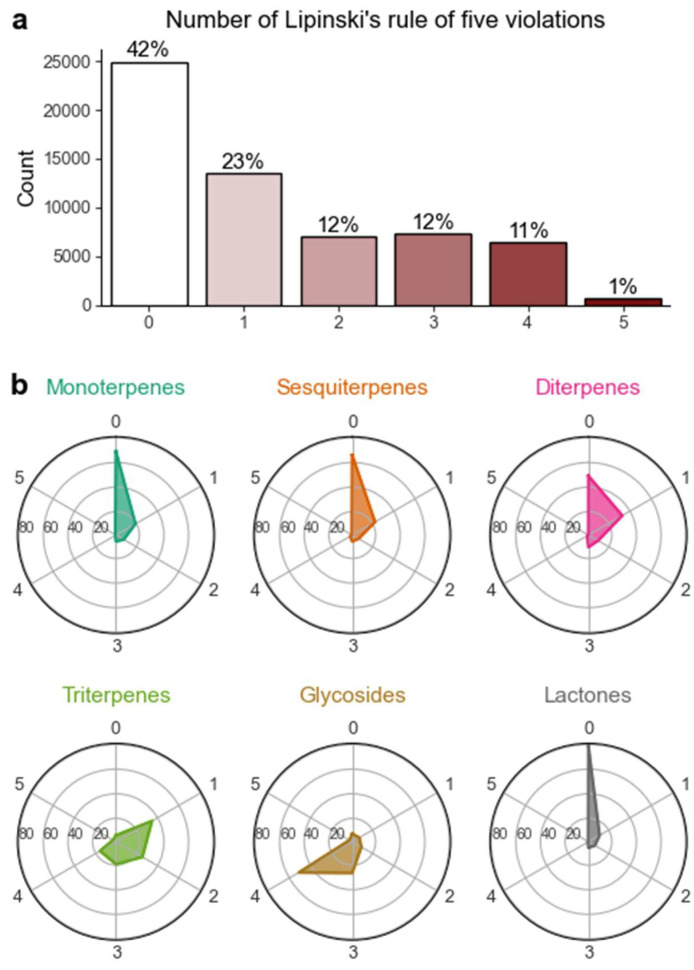
(**a**) Number of violations of Lipinski’s rule of five in the terpene chemical space, for all terpenes, and (**b**) across distinct subclasses; outer values correspond to the number of violations and inner values the percentage of a given number of violations.

After this, the authors were interested in quantifying how many molecules violated any of the postulated Lipinski rules. As shown in Figure 7, 41.6% of terpenes do not present any violation. While the most common deviation is 1 violation (22.8%), it is of note that there is an almost equivalent count of molecules with 2, 3 and 4 violations, which cumulatively account for 35% of all molecules. The authors further investigated the number of violations across different terpenes subclasses. As seen in Figure 7, there are remarkable differences. 

Monoterpenes and sesquiterpenes present a similar and nearly superimposable profile and distribution of number of violations. Triterpenes are unique in the way they present the lowest percentage of compounds with 0 violations, only 5.2%, while nearly 60% present 1 or 2 violations. Equally unique are terpene glycosides, which include over 50% of molecules with 4 violations, a unique trait, considering that the second class in this category is sesquiterpenes, with only 1.9%. Likewise, terpene lactones are a rather particular class, as nearly 80% of the molecules in this class present no violations of Lipinski’s rule of five. The results presented so far provide a valuable source of information that can be used to select the terpene subclasses that present a chemical profile more compatible to future drug development frameworks.

### 2.2. Clustering the Terpene Chemical Space

In high dimensional data, clustering can be an effective method to make sense of heterogeneous data. In the case of chemical information, it can be used to identify the most important physicochemical parameters that allow grouping different families of molecules. In the specific case of terpenes, this can be useful where a new chemical entity is found, and its sorting in a given class is challenging owing to inexistent biosynthetic data, mostly in the case of molecules with an odd number of carbons.

In the case of terpenes, the authors were interested in studying if their physicochemical parameters could *per se* help position a given molecule in a particular subclass. To this end, and given the high dimensionality of the data, the authors employed several dimensionality reduction methods, namely PCA, UMAP, t-SNE, FastICA and Kernel PCA. After this, the authors tried to cluster the data, both the original data along with the reduced form, by k-means and hierarchical clustering and benchmarked the results.

To evaluate the quality of the clustering, the authors considered the following metrics. The homogeneity score (shorthand: Homo) measures if each cluster includes only the datapoints that are members of a single class. The completeness score (shorthand: Compl) shows if all datapoints of a given class are members of the same cluster. The harmonic mean between the homogeneity and completeness scores is computed by v-measure (shorthand: V-meas) [11]. The Rand index [12] shows the similarity between two label assignments, and the adjusted Rand index (shorthand: ARI) is a version of the Rand index that corrects for chance. The agreement between two label assignments, ignoring permutations, is computed by mutual information. Here, we used adjusted mutual information (shorthand: AMI), which is normalized against the chance version of it. The above-mentioned measures fall within the range 0 to 1, with score 1 meaning perfectly labelling. 

The silhouette coefficient [13], however, ranges from –1 to 1 and measures how similar a datapoint is to its own cluster rather than other clusters. Higher silhouette values show that a datapoint is highly similar to its own cluster and poorly similar to neighbouring clusters. 

In addition to the mentioned metrics, the authors measured the execution time of different methods running on Apple M1 CPU.

Supplementary Tables S4 and S5 demonstrate, in detail, the results of carrying out k-means and agglomerative clustering on imbalanced and balanced datasets, with and without applying dimensionality reduction methods with the parameters shown in Supplementary Table S3.

Of those results, the best ones are shown in Table 1. 

In the case of k-means on imbalanced data, PCA provides metric values ≤ 0.33 with 11 principal components, which are low-performing values, as they should be as close to 1 as possible. When compared to PCA, UMAP (n_neighbors = 45, min_dist = 0.1, Supplementary Table S3) did not provide any substantial increase in performance, except for silhouette, which yielded an increase of 0.21. t-SNE did not provide any improvement over UMAP, despite the multiple settings tried; the same was true for FastICA and Kernal PCA, except for the silhouette metric. Kernal PCA afforded the best silhouette, 0.50, which is still low and, hence, not suitable for our clustering purposes. Considering these results, the authors wondered if the reason for this poor performance could be related to some degree of imbalance in the data.

To account for this, the authors applied random oversampling (ROS) for data balancing, after which all algorithms were run once more. Note that since it was important for the authors not to generate synthetic data, they did not employ synthetic sampling approaches. As shown in the table, data balancing did not improve the results; the three subclasses we fed to the clustering algorithm, i.e., “Triterpenes”, “Diterpenes” and “Monoterpenes”, had the occurrences of 13245, 11814 and 5115, respectively.

The same procedure as above was repeated by hierarchical (agglomerative) clustering. Table 1 shows that applying UMAP (UMAP7), before clustering, provides the best results when the authors cluster on imbalanced data. These results are almost the same as the ones obtained by UMAP7 plus k-means, with the difference that the latter is 70% faster. Similar to before, data balancing could not help improve the results. Therefore, the best results are obtained by first applying UMAP7 to reduce the dimensionality of the data (the original imbalanced one) and then k-means to cluster it. Note that even these methods could not provide a satisfactory result in clustering the three target subclasses.

### 2.3. Classification

The authors were also interested in assessing the suitability of this data for classification purposes. This is an important task, as it may help classify novel molecules to a given terpene subclass in cases where biosynthetic studies are not yet available.

Several classification methods were fitted, namely LightGBM, kNN, random forests, Gaussian naïve Bayes and MLP, with their default parameters in scikit-learn on the terpenes training data, which was obtained after the data cleansing described in the “Methods” section; then, we validated them by the 5-fold cross-validation technique.

To evaluate the classification methods, we measured the following metrics, which range from 0 to 1, with the values closer to 1 showing better performance. Considering the fact that the target feature, i.e., “chemicalSubClass”, is imbalanced, the authors used the weighted version of the metrics.

Precision shows if the classifier is able to not label negative samples as positive. Recall shows if the classifier can find all the positive samples. The F1 score is calculated by taking the harmonic mean of the precision and recall. Balanced accuracy is the average of recall scores for each class. Area under curve of the receiver operating characteristic (shorthand: ROC-AUC) measures the performance, as the discrimination threshold of the classifier varies. The authors set the “multi_class” parameter in the implementation as “ovo”, which stands for one-vs-one and calculates the average area under curve (AUC) for all pairs of classes.

Supplementary Table S6 shows the results of cross-validation on the terpenes training data. Since LightGBM and random forest performed similarly in producing the best results, we optimized hyperparameters of both methods, employing randomized search, and cross-validated them again on the training data. Carrying out with the optimized hyperparameters (Supplementary Table S7), it is shown in Supplementary Table S8 that LightGBM performed better, however slightly, while running 4.7 times faster. It reaches to 91% balanced accuracy and 99% ROC-AUC on the validation data. Based on what we described, LightGBM was chosen to classify the terpenes subclasses.

**Figure 8:**
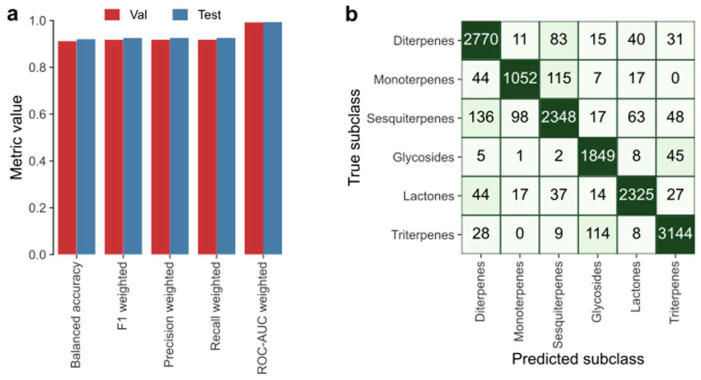
LightGBM applied on the terpenes data, by the parameters described in Supplementary Table S7. (**a**) Cross-validation and test results and (**b**) confusion matrix for the test data.

In Figure 8, the evaluation results of applying LightGBM on the terpenes data is depicted. Figure 8a shows that at least 0.91 was approached (out of 1.00) by all the considered metrics; for instance, balanced accuracy and weighted F1 score on the test data are 0.92 and 0.93, respectively. 

Figure 8b shows the confusion matrix obtained by applying LightGBM on the test data, from which we could calculate the weighted metrics shown in Figure 8a, since it denotes true positives, false positives, true negatives and false negatives for each subclass. Given the positive results obtained, the authors succeeded in applying a classifier capable of classification of terpene molecules on the grounds of their physicochemical properties.

## 3. Methods

### 3.1. Data Collection

The authors started with the COCONUT database available at https://coconut.naturalproducts.net (database version from 21 April 2021). The dataset had the original shape of 401,624 entries in 1554 columns. Initial data assessment showed that in most of the columns, more than 70% of entries were NULL, the reason for which those columns were dropped. In addition, there were a few unique identifiers, such as “_id” and “coconut_id”, which had no predictive or informative value. Such variables were dropped, thus originating the dataset used, with 401,624 entries and 45 variables, which are described in Supplementary Table S1. We then selected only the entries that belonged to the SuperClass “Lipids and lipid-like molecules” ( Supplementary Table S2) and further filtered only the molecules that belonged to one of the following SubClasses: “Diterpenes”, “Sesquiterpenes”, “Monoterpenes”, “Polyterpenes”, “Sesquaterpenes”, “Sesterterpenes”, “Terpene glycosides”, “Terpene lactones” and “Triterpenes”. After this selection, the resulting dataset included 59,833 molecules.

### 3.2. Data Cleansing

To preprocess the data, multiple steps were taken. First, the authors handled categorical features, including “textTaxa”, “bcutDescriptor”, “chemicalClass”, “chemicalSubClass”, “chemicalSuperClass” and “directParentClassification”. To encode the “textTaxa” feature, we created four new columns for “plants”, “marine”, “bacteria” and “fungi” taxonomy in a way that, for each molecule having either of those values, a 1 was inserted to the corresponding column, and 0 otherwise. The “bcutDescriptor” feature included arrays of six float numbers; we split and expanded those into six separate columns. Since all terpenes share the same “chemicalClass” and “chemicalSuperClass”, we did not consider them for further processes. “chemicalSubClass” was the target, so we did not encode it. The “directParentClassification” feature, containing 111 values, was simply encoded by the integers 0 to 110.

For further processing, the dataset was split into 75% training and 25% testing data. Next, we imputed the data using the median strategy, i.e., replaced missing values with the median of not-null training values in each column. Then, in order to scale the data, the authors standardized it by centring the data around the mean (removing the mean) with a unit variance. Note that the features with binary values, such as “contains_sugar”, do not need to be scaled.

### 3.3. Clustering

For clustering, the authors applied k-means and agglomerative clustering methods on the dataset, as a whole, and also on the dimensionality reduced form of it. k-means is a widely used method that partitions the data into k clusters in a way that each datapoint belongs to the cluster with the closest centroid (cluster centre). Agglomerative clustering is another popular method that performs hierarchical clustering, in which, starting from each datapoint that forms one cluster, and pairs of clusters are recursively merged based on linkage distance. k-means and agglomerative clustering follow different approaches, i.e., “top-down” and “bottom-up” approaches, respectively. Clustering has been widely used in the field of medicine and biomedical sciences for applications such as selecting new candidate drugs for lung cancer [14], molecular descriptor analysis [15] or clustering the gene profiles of distinct types of cancer [16].

Several dimensionality reduction algorithms were employed for our purpose, namely PCA, FastICA [17], Kernel PCA [18], t-SNE [19] and UMAP [20]. PCA (principal component analysis) changes the basis of data by computing principal components in order to project the high-dimensional data into a lower dimensional space. FastICA (fast independent component analysis) is an efficient algorithm that finds an orthogonal rotation of prewhitened data, with the statistical independence assumption. Kernel PCA (kernel principal component analysis) is as an extension of PCA that employs kernels, such as RBF (radial basis function), to reduce the dimensionality of data, non-linearly. t-SNE (t-distributed stochastic neighbour embedding) is a non-linear technique that employs stochastic neighbour embedding to project each datapoint in a high-dimensional space into a location in a 2- or 3-dimensional space. UMAP (uniform manifold approximation and projection) is a manifold learning method that is based on Riemannian geometry and algebraic topology; its output is visually similar to t-SNE, but it does not have computational limitations on embedding dimension. In the field of biomedical sciences, PCA is the most widely used method [21]; however, UMAP has been receiving significant attention since its introduction [22].

To carry out the above-mentioned methods, the authors used “scikit-learn” and “umap” Python libraries that can be accessed at https://github.com/scikit-learn/scikit-learn (accessed on 30 September 2021) and https://github.com/lmcinnes/umap (accessed on 30 September 2021), respectively. In addition, the parameters used are listed in Supplementary Table S3. Note that we performed clustering on the data belonging to the “Triterpenes”, “Diterpenes” and “Monoterpenes” subclasses.

### 3.4. Classification

The authors carried out multiple methods, namely, Light gradient boosting machine (LightGBM) [23], k-nearest neighbours (kNN) [24], random forests [25], Gaussian naïve Bayes [26] and Multilayer perceptron (MLP), to classify six subclasses of “Monoterpenes”. “Triterpenes”, “Diterpenes”, “Sesquiterpenes”, “Terpene lactones” and “Terpene glycosides”. LightGBM is a distributed gradient boosting framework that works based on decision tree algorithms. In the kNN method, the class membership of an object is determined by the most common class between its k nearest neighbours. Random forests are an ensemble learning method, in which different decision tree classifiers are fit on various sub-samples of the data. Gaussian naïve Bayes is based on the Bayes theorem and follows the Gaussian normal distribution. Multilayer perceptron is a feedforward neural network with at least three layers of input, hidden and output, that uses backpropagation for training and uses a non-linear activation function. Among the mentioned algorithms, LightGBM and random forest are both ensemble methods based on decision trees, with the difference that the first one is categorized as a boosting algorithm, but the second one is a bagging (bootstrap aggregating) method. In a different point of view, Gaussian NB and MLP are parametric models, meaning that they perform learning with parameters of fixed size and are independent of the size of the dataset, while the others, i.e., LightGBM, kNN and random forest, are non-parametric algorithms. Note that for the implementation, we used the “scikit-learn” and “lightgbm” (https://github.com/microsoft/LightGBM) Python libraries, accessed on 30 September 2021.

## 4. Conclusions

In this work, the authors have exploited the significant growth in terms of data accessibility in the field of natural products to better define the chemical space of terpenes. Information on a subclass-by-subclass basis of some of the most important parameters for drug development is also provided and further contributes to the phytochemical knowledge of this group of natural molecules.

The authors also tried to apply a number of dimensionality reduction and clustering algorithms to assess the suitability of the available data to this end. The results were not as good as desired, which calls for increasingly well-annotated data on natural products and new methods.

This work also shows that several algorithms are able to successfully classify terpenes solely using their physicochemical descriptors and to provide information on the performance of said algorithms. The results provided here can be useful for further studies on this class of natural products, including those involving the selection of molecules with favourable characteristics from a pharmaceutical point of view.

## Figures and Tables

**Table 1:** Clustering the original terpenes data along with dimensionality-reduced form of it, in both imbalanced and balanced forms. Parameters are described in Supplementary Table S3. * Less than 1 s.

Dim Reduce	Time (s)	Homo	Compl	V-meas	ARI	AMI	Silhouette
*k*-means on imbalanced data							
original	0 *	0.30	0.32	0.31	0.23	0.31	0.19
PCA0	0 *	0.32	0.33	0.33	0.25	0.33	0.21
UMAP7	19	0.39	0.40	0.40	0.32	0.40	0.42
TSNE6	45	0.21	0.20	0.21	0.19	0.21	0.39
FastICA	0 *	0.23	0.25	0.24	0.16	0.24	0.46
Kernel PCA	72	0.24	0.23	0.23	0.20	0.23	0.50
*k*-means on balanced data							
original	0 *	0.27	0.31	0.29	0.25	0.29	0.21
PCA1	0 *	0.27	0.30	0.29	0.24	0.29	0.40
UMAP7	26	0.11	0.11	0.11	0.12	0.11	0.36
TSNE6	65	0.24	0.24	0.24	0.24	0.24	0.38
FastICA	0 *	0.19	0.22	0.20	0.13	0.20	0.47
Kernel PCA	1353	0.25	0.25	0.25	0.24	0.25	0.45
Agglomerative clustering on imbalanced data							
original	23	0.32	0.32	0.32	0.27	0.32	0.13
PCA0	16	0.32	0.32	0.32	0.28	0.32	0.16
UMAP7	32	0.40	0.40	0.40	0.34	0.40	0.41
TSNE8	127	0.21	0.20	0.21	0.24	0.21	0.32
FastICA	11	0.21	0.20	0.20	0.18	0.20	0.34
Kernel PCA	83	0.17	0.17	0.17	0.13	0.17	0.40
Agglomerative clustering on balanced data							
original	56	0.15	0.17	0.16	0.11	0.16	0.21
PCA0	37	0.27	0.32	0.29	0.25	0.29	0.22
UMAP7	51	0.10	0.11	0.10	0.10	0.10	0.34
TSNE6	93	0.30	0.33	0.32	0.26	0.32	0.32
FastICA	26	0.17	0.20	0.18	0.12	0.18	0.41
Kernel PCA	1381	0.28	0.30	0.29	0.28	0.29	0.40

Results highlight quantitative diferences using a color grad.

## Data Availability

The data presented in this study are openly available in https://github.com/smortezah/napr (accessed on 19 January 2023).

## Supplementary Materials

The following supporting information can be downloaded at: https://www.mdpi.com/article/10.3390/ph16020202/s1, Supplementary Table S1. Features of the COCONUT database that were used and their description. Supplementary Table S2. Distribution of Lipids and lipid-like molecules. Supplementary Table S3. Parameters of clustering and dimensionality reduction methods run in the experiments. Supplementary Table S4. k-means clustering on the original data along with dimensionality reduced form of it, running on imbalanced and balanced data. Supplementary Table S5. Agglomerative clustering on the original data and dimensionality reduced data, running on imbalanced and balanced data. Supplementary Table S6. Cross-validation of classification methods, without optimized hyperparameters, on the terpenes training data. Accuracy is “balanced” and the other metrics, except time, are “weighted”. Supplementary Table S7. Hyperparameter optimization of LightGBM and random forest running on the terpenes training data. Supplementary Table S8. Cross-validation of LightGBM and random forest methods with optimized hyperparameters on the terpenes training data. Accuracy is “balanced” and the other metrics, except time, are “weighted”.
