# Supplementary material for: The Chemical Space of Terpenes: Insights from Data Science and AI
Source: Pharmaceuticals (Basel). 2023 Jan 29;16(2):202. doi: 10.3390/ph16020202 (PMC9961535; doi:10.3390/ph16020202)
Supplement: Supplementary file 1 [file pharmaceuticals-16-00202-s001.zip › pharmaceuticals-2054179-supplementary.pdf]

Supplementary material

Supplementary Table S1 - Features of the COCONUT database that were used and their description.

Feature	Description
alogp (Ghose-Crippen LogKow)	logP (Ghose-Crippen LogKow)
alogp2	alogP squared
amralogp	AMR - molar refractivity
apol	Sum of the atomic polarizabilities
bcutDescriptor	BCUT decriptor (Eigenvalue based)
bpol	BPol descriptor
cas	CAS code
chemicalClass	Chemical class of the NP (computed with ClassyFire)
chemicalSubClass	Chemical subclass of the NP (computed with ClassyFire)
chemicalSuperClass	Chemical superclass of the NP (computed with ClassyFire)
contains_ring_sugars	Bolean for the presence of circular sugars
contains_sugar	Bolean for the presence of linear sugars
directParentClassification	Classification of parent molecule
eccentricConnectivityIndexDescriptor	Eccentric Connectivity Index Descriptor
fmfDescriptor	fmf Descriptor
fragmentComplexityDescriptor	Fragment Complexity Descriptor
fsp3	Fractional CSP3 Descriptor (non-flatness of a molecule)
hBondAcceptorCount	Hydrogen bond acceptor count
hBondDonorCount	Hydrogen bond donor count
heavy_atom_number	Number of heavy atoms
hybridizationRatioDescriptor	Hybridization Ratio Descriptor (fraction of sp3 carbons to sp2 carbons)
iupac_name	IUPAC name
kappaShapeIndex1	First kappa shape index
kappaShapeIndex2	Second kappa shape index
kappaShapeIndex3	Third kappa shape index
lipinskiRuleOf5Failures	Number of Lipinski Rule of 5 violations
manholdlogp	LogP descriptor (Mannhold version)
max_number_of_rings	Maximal number of rings
min_number_of_rings	Minimal number of rings
molecular_formula	Molecular formula
molecular_weight	Molecular weight
name	Name of the molecule
npl_noh_score	Natural product-likeness score without taking into account any hydrogen in the molecular structure
npl_score	Natural product-likeness score
npl_sugar_score	NP-likeness score computed on the glycosylated molecule
number_of_carbons	Number of carbons
number_of_nitrogens	Number of nitrogens
number_of_oxygens	Number of oxygens
numberSpiroAtoms	Number of spiro atoms
directParentClassification	Direct parent in the chemical ontology (computed with Classyfire)
petitjeanNumber	Petitjean Number
petitjeanShapeTopo	Petitjean geometrical shape index
smiles	SMILES notation
sugar_free_heavy_atom_number	Number of heavy atoms of the deglycosylated moiety
sugar_free_smiles	SMILES of the deglycosylated moiety

sugar_free_total_atom_number	Total atom number of the deglycosylated moiety
textTaxa	List of organisms producing the NP in text form
topoPSA	Topological polar surface area descriptor
total_atom_number	Total atom number
tpsaEfficiency	Fractional polar surface area descriptor
vertexAdjMagnitude	Vertex adjacency information
weinerPathNumber	Wiener Path Number
weinerPolarityNumber	Wiener Polarity Number

Supplementary Table S2 - Distribution of Lipids and lipid-like molecules.

Class	Count	Out of total
Triterpenoids	13,245	13.411%
Diterpenoids	11,814	11.962%
Sesquiterpenoids	10,815	10.950%
Terpene lactones	9580	9.700%
Terpene glycosides	7727	7.824%
Monoterpenoids	5115	5.179%
Steroidal glycosides	4541	4.598%
Fatty acids and conjugates	3177	3.217%
Fatty alcohols	2848	2.884%
Steroid lactones	2641	2.674%
Fatty acid esters	2182	2.209%
Fatty acyl glycosides	1380	1.397%
Sesterterpenoids	1326	1.343%
Pregnane steroids	1301	1.317%
Bile acids, alcohols and derivatives	1283	1.299%
Glycerophosphocholines	1243	1.259%
Glycerophosphoethanolamines	1161	1.176%
Steroid esters	1099	1.113%
Androstane steroids	882	0.893%
Oxosteroids	881	0.892%
Ergostane steroids	853	0.864%
Diradylglycerols	822	0.832%
Hydroxysteroids	818	0.828%
Cholestane steroids	789	0.799%
Eicosanoids	785	0.795%
Stigmastanes and derivatives	736	0.745%
Glycosphingolipids	712	0.721%
Quinone and hydroquinone lipids	711	0.720%
Fatty alcohol esters	710	0.719%
Tetraterpenoids	676	0.684%
Cycloartanols and derivatives	643	0.651%
Fatty amides	527	0.534%
Lineolic acids and derivatives	494	0.500%
Glycerophosphoinositols	445	0.451%
Triradylcglycerols	440	0.446%
Steroidal alkaloids	381	0.386%
Fatty acyl thioesters	280	0.284%
Estrane steroids	264	0.267%
Hopanoids	244	0.247%
Glycosylglycerols	243	0.246%
Glycerophosphoinositol phosphates	237	0.240%
Ecdysteroids	228	0.231%
Cucurbitacins	212	0.215%
Ceramides	179	0.181%
Sesquaterpenoids	163	0.165%
Fatty aldehydes	157	0.159%
Monoradylglycerols	132	0.134%
Glycerophosphoglycerols	130	0.132%
Glycerophosphoserines	116	0.117%
Phosphosphingolipids	107	0.108%
Glycerophosphoglycerophosphates	106	0.107%
Glycerophosphoglycerophosphoglycerols	101	0.102%

Gorgostanes and derivatives	99	0.100%
Vitamin D and derivatives	93	0.094%
Polyprenols	91	0.092%
Steroid acids	86	0.087%
Azasteroids and derivatives	84	0.085%
Retinoids	76	0.077%
Acyltrehaloses	58	0.059%
Polyterpenoids	48	0.049%
Delta-7-steroids	41	0.042%
Furostanes and derivatives	40	0.041%
CDP-glycerols	40	0.041%
Glycerophosphates	40	0.041%
Furospirostanes and derivatives	37	0.037%
Physalins and derivatives	35	0.035%
Oxasteroids and derivatives	33	0.033%
Sulfated steroids	32	0.032%
Delta-5-steroids	27	0.027%
17-furanylsteroids and derivatives	25	0.025%
Polyprenylphenols	22	0.022%
Glycerol vinyl ethers	19	0.019%
5,6-epoxysteroids	17	0.017%
Glycerol ethers	11	0.011%
Isoprenoid phosphates	11	0.011%
C24-propyl sterols and derivatives	6	0.006%
Halogenated steroids	3	0.003%
Delta-1,4-steroids	2	0.002%
Glycerophosphonocholines	2	0.002%
Lysobisphosphatidic acids	1	0.001%
Semilysobisphosphatidic acids	1	0.001%
Glycerophosphonoethanolamines	1	0.001%
Glycero-3-pyrophosphates	1	0.001%
Phosphonosphingolipids	1	0.001%

Supplementary Table S3 - Parameters of clustering and dimensionality reduction methods run in the experiments.

**Method**	**Parameters**
**Clustering**	
k-means	n_clusters=9, n_init=4
Agglomerative	n_clusters=9, linkage='ward'
**Dimensionality reduction**	
PCA0	n_components=0.9 (automatically choose the number of components that preserve 90% of variance. In our case, it is 11.)
PCA1	n_components=2
UMAP0	n_neighbors=5, min_dist=0.01
UMAP1	n_neighbors=5, min_dist=0.1
UMAP2	n_neighbors=5, min_dist=0.4
UMAP3	n_neighbors=13, min_dist=0.01
UMAP4	n_neighbors=13, min_dist=0.1
UMAP5	n_neighbors=13, min_dist=0.4
UMAP6	n_neighbors=45, min_dist=0.01
UMAP7	n_neighbors=45, min_dist=0.1
UMAP8	n_neighbors=45, min_dist=0.4
TSNE0	perplexity=5, n_iter=300
TSNE1	perplexity=5, n_iter=900
TSNE2	perplexity=5, n_iter=1300
TSNE3	perplexity=20, n_iter=300
TSNE4	perplexity=20, n_iter=900
TSNE5	perplexity=20, n_iter=1300
TSNE6	perplexity=50, n_iter=300
TSNE7	perplexity=50, n_iter=900
TSNE8	perplexity=50, n_iter=1300
FastICA	tol=0.1
Kernel PCA	kernel='cosine'

Supplementary Table S4 - *k*-means clustering on the original data along with dimensionality reduced form of it, running on imbalanced and balanced data. Parameters are described in Supplementary Table S3.

Dim reduce	Time (s)	Homo	Compl	V-meas	ARI	AMI	Silhouette
**Imbalanced data**							
original	0*	0.30	0.32	0.31	0.23	0.31	0.19
PCA0	0*	0.32	0.33	0.33	0.25	0.33	0.21
PCA1	0*	0.27	0.28	0.28	0.19	0.28	0.39
UMAP0	14	0.01	0.01	0.01	0.01	0.01	0.37
UMAP1	10	0.01	0.01	0.01	0.01	0.01	0.34
UMAP2	10	0.04	0.03	0.03	0.03	0.03	0.36
UMAP3	13	0.01	0.01	0.01	0.01	0.01	0.35
UMAP4	13	0.05	0.04	0.04	0.03	0.04	0.35
UMAP5	12	0.12	0.11	0.11	0.10	0.11	0.37
UMAP6	19	0.35	0.36	0.36	0.29	0.35	0.46
UMAP7	19	0.39	0.40	0.40	0.32	0.40	0.42
UMAP8	19	0.38	0.39	0.38	0.31	0.38	0.38
TSNE0	36	0.01	0.01	0.01	0.01	0.01	0.37
TSNE1	69	0.01	0.01	0.01	0.01	0.01	0.36
TSNE2	96	0.02	0.02	0.02	0.02	0.01	0.38
TSNE3	39	0.08	0.07	0.08	0.07	0.07	0.38
TSNE4	76	0.07	0.06	0.06	0.06	0.06	0.35
TSNE5	100	0.07	0.07	0.07	0.04	0.06	0.37
TSNE6	45	0.21	0.20	0.21	0.19	0.21	0.39
TSNE7	89	0.20	0.19	0.19	0.18	0.19	0.39
TSNE8	115	0.20	0.19	0.19	0.18	0.19	0.38
FastICA	0*	0.23	0.25	0.24	0.16	0.24	0.46
Kernel PCA	72	0.24	0.23	0.23	0.20	0.23	0.50
**Balanced data**							
original	0*	0.27	0.31	0.29	0.25	0.29	0.21
PCA0	0*	0.27	0.31	0.29	0.25	0.29	0.24
PCA1	0*	0.27	0.30	0.29	0.24	0.29	0.40
UMAP0	100	0.02	0.02	0.02	0.02	0.02	0.33
UMAP1	103	0.02	0.02	0.02	0.02	0.02	0.33
UMAP2	105	0.02	0.02	0.02	0.02	0.02	0.34
UMAP3	22	0.02	0.02	0.02	0.02	0.02	0.32
UMAP4	22	0.06	0.06	0.06	0.07	0.06	0.33
UMAP5	22	0.01	0.01	0.01	0.01	0.01	0.34
UMAP6	26	0.07	0.08	0.07	0.09	0.07	0.37
UMAP7	26	0.11	0.11	0.11	0.12	0.11	0.36
UMAP8	26	0.02	0.02	0.02	0.02	0.02	0.35
TSNE0	56	0.01	0.01	0.01	0.01	0.01	0.36
TSNE1	109	0.02	0.02	0.02	0.02	0.02	0.36
TSNE2	141	0.01	0.01	0.01	0.02	0.01	0.36
TSNE3	61	0.15	0.15	0.15	0.16	0.15	0.37
TSNE4	111	0.09	0.09	0.09	0.09	0.09	0.36

TSNE5	142	0.14	0.14	0.14	0.15	0.14	0.36
TSNE6	65	0.24	0.24	0.24	0.24	0.24	0.38
TSNE7	123	0.19	0.19	0.19	0.20	0.19	0.37
TSNE8	170	0.21	0.21	0.21	0.21	0.21	0.37
FastICA	0*	0.19	0.22	0.20	0.13	0.20	0.47
Kernel PCA	1353	0.25	0.25	0.25	0.24	0.25	0.45

* Less than 1 second.

Supplementary Table S5 - Agglomerative clustering on the original data and dimensionality reduced data, running on imbalanced and balanced data. Parameters are described in Supplementary Table S3.

Dim reduce	Time (s)	Homo	Compl	V-meas	ARI	AMI	Silhouette
**Imbalanced data**							
original	23	0.32	0.32	0.32	0.27	0.32	0.13
PCA0	16	0.32	0.32	0.32	0.28	0.32	0.16
PCA1	13	0.20	0.23	0.22	0.13	0.22	0.38
UMAP0	25	0.02	0.02	0.02	0.02	0.02	0.28
UMAP1	21	0.02	0.02	0.02	0.02	0.02	0.31
UMAP2	21	0.05	0.05	0.05	0.05	0.05	0.28
UMAP3	25	0.05	0.05	0.05	0.03	0.05	0.31
UMAP4	24	0.09	0.10	0.09	0.07	0.09	0.30
UMAP5	23	0.17	0.18	0.18	0.14	0.18	0.30
UMAP6	32	0.36	0.35	0.35	0.31	0.35	0.45
UMAP7	32	0.40	0.40	0.40	0.34	0.40	0.41
UMAP8	32	0.40	0.39	0.40	0.34	0.40	0.37
TSNE0	46	0.02	0.02	0.02	0.02	0.02	0.31
TSNE1	78	0.01	0.01	0.01	0.01	0.01	0.34
TSNE2	108	0.02	0.02	0.02	0.02	0.02	0.33
TSNE3	50	0.07	0.07	0.07	0.08	0.07	0.34
TSNE4	86	0.05	0.06	0.05	0.05	0.05	0.31
TSNE5	110	0.04	0.03	0.03	0.04	0.03	0.33
TSNE6	56	0.15	0.16	0.16	0.14	0.15	0.32
TSNE7	100	0.11	0.10	0.11	0.10	0.11	0.34
TSNE8	127	0.21	0.20	0.21	0.24	0.21	0.32
FastICA	11	0.21	0.20	0.20	0.18	0.20	0.34
Kernel PCA	83	0.17	0.17	0.17	0.13	0.17	0.40
**Balanced data**							
original	56	0.15	0.17	0.16	0.11	0.16	0.21
PCA0	37	0.27	0.32	0.29	0.25	0.29	0.22
PCA1	24	0.18	0.22	0.20	0.13	0.20	0.40
UMAP0	122	0.02	0.02	0.02	0.02	0.02	0.29
UMAP1	132	0.02	0.02	0.02	0.02	0.02	0.31
UMAP2	127	0.02	0.02	0.02	0.02	0.02	0.28
UMAP3	54	0.01	0.01	0.01	0.01	0.01	0.29
UMAP4	46	0.04	0.04	0.04	0.04	0.04	0.28
UMAP5	45	0.02	0.02	0.02	0.01	0.02	0.29
UMAP6	55	0.05	0.05	0.05	0.05	0.05	0.32
UMAP7	51	0.10	0.11	0.10	0.10	0.10	0.34
UMAP8	49	0.05	0.05	0.05	0.05	0.05	0.29
TSNE0	80	0.04	0.04	0.04	0.04	0.04	0.33
TSNE1	133	0.02	0.02	0.02	0.02	0.02	0.32
TSNE2	161	0.01	0.01	0.01	0.01	0.01	0.33
TSNE3	84	0.12	0.12	0.12	0.09	0.12	0.33
TSNE4	132	0.17	0.19	0.18	0.15	0.18	0.29

TSNE5	165	0.09	0.09	0.09	0.07	0.09	0.28
TSNE6	93	0.30	0.33	0.32	0.26	0.32	0.32
TSNE7	144	0.13	0.13	0.13	0.14	0.13	0.34
TSNE8	191	0.16	0.16	0.16	0.16	0.16	0.32
FastICA	26	0.17	0.20	0.18	0.12	0.18	0.41
Kernel PCA	1381	0.28	0.30	0.29	0.28	0.29	0.40

Supplementary Table S6 - Cross-validation of classification methods, without optimized hyperparameters, on the terpenes training data. Accuracy is "balanced" and the other metrics, except time, are "weighted".

Method	Time (s)	Accuracy	F1	Precision	Recall	ROC-AUC
LightGBM	16	0.90	0.90	0.90	0.90	0.99
*k*NN	75	0.84	0.85	0.85	0.85	0.96
Random forest	87	0.89	0.91	0.91	0.91	0.99
Gaussian NB	88	0.50	0.44	0.49	0.46	0.84
MLP	184	0.85	0.86	0.86	0.86	0.98

Supplementary Table S7 - Hyperparameter optimization of LightGBM and random forest running on the terpenes training data.

**Method**	**Hyperparam**	**Search space**	**Optimized hyperparam**
LightGBM	learning_rate	0.005, 0.01, 0.05, 0.1, 0.5, 0.75, 1	0.5
	n_estimators	25, 50, 100, 150, 200	150
	num_leaves	10, 15, 25, 31, 35	31
	boosting_type	‘gbdt’, ‘dart’, ‘goss’	’dart’
	colsample_bytree	0.25, 0.5, 0.75, 1	0.5
	subsample	0.25, 0.5, 0.75, 1	1
	reg_alpha	0.25, 0.5, 1, 1.2	1
	reg_lambda	0.25, 0.5, 1, 1.2	0.5
Random forest	bootstrap	True, False	False
	max_depth	30, 60, 90, None	90
	min_samples_leaf	1, 2, 4	1
	min_samples_split	2, 5, 10	10
	n_estimators	100, 500, 1000, 1500	1000

Supplementary Table S8 - Cross-validation of LightGBM and random forest methods with optimized hyperparameters on the terpenes training data. Accuracy is "balanced" and the other metrics, except time, are "weighted".

Method	Time (s)	Accuracy	F1	Precision	Recall	ROC-AUC
LightGBM	34	0.91	0.92	0.92	0.92	0.99
Random forest	160	0.90	0.91	0.91	0.91	0.99
